# Smart betas, return models and the tangency portfolio weights

**DOI:** 10.1371/journal.pone.0305736

**Published:** 2024-06-25

**Authors:** Jan Lennartsson, Claes Ekman

**Affiliations:** Andra AP-fonden (AP2), Göteborg, Sweden; University of Bari Aldo Moro: Universita degli Studi di Bari Aldo Moro, ITALY

## Abstract

In this paper, we analytically derive closed-form expressions for the tangency portfolio weights: the fully invested portfolio that maximizes the expected return over the risk-free rate, relative to the volatility of the portfolio return. We explicitly derive this portfolio from a range of underlying return models and show examples where it coincides with different well-known smart beta products. Specifically, we find the closed-form expression for the tangency portfolio weights for a return model with compound symmetric correlation matrix. We also deduce the tangency portfolio weights for the CAPM return model and illustrate in a case study that the estimated tangency portfolio weights may distinctly deviate from the market value weighted portfolio. Furthermore, we show that depending on the return model, the tangency portfolio weights may take a diverse set of shapes; from very diversified to highly concentrated portfolios.

## Introduction

Modern portfolio theory was initiated by Harry Markowitz in his seminal paper, Portfolio Selection [1]. There, Markowitz introduced the concept of relating the securities’ expected return to their inherent risk, where risk was quantified in terms of volatility of the security returns. Subsequently, according to the risk-return framework, the relation of portfolios’ expected return and risk was taken into consideration. The investor wants the portfolio to feature high expected return and low risk. Further, Markowitz laid out that a rational investor is an investor who seeks the largest expected return for the equal amount of risk. In Markowitz’s terminology, this gives rise to the efficient frontier, the portfolios that maximize the expected return for each level of risk.

Later, James Tobin [2] developed the theory of portfolio construction by adding a risk-free constituent to the set of investable securities. Further, he divided the problem of finding an optimal portfolio into two separate subproblems; first allocating between the risky assets and then combining them with an appropriate amount of the risk-free security. A consequence of Tobin’s results, see e.g. [3, 4] or [5], is that all rational investors will feature a portfolio consisting of one part the risk-free investment and one part risky securities that maximizes the ratio of expected return above the risk-free rate, divided by the volatility of the returns of the portfolio. The second part of that portfolio is known as the tangency portfolio weights or using the shortened phrase, tangency portfolio. Furthermore, an effect of Tobin’s separation theorem is that, independent of the level of risk, the relative allocation of funds between the non risk-free assets should be identical for all investors, given that they share the same underlying return model. This is used as a strong argument by proponents for allocating funds aligned with the market value-weighted portfolio. Given that all investors are rational and feature the same model for returns, then all investors seek to allocate funds among risky assets identically. Hence, all rational portfolio managers invest proportionally to the tangency portfolio weights, and if all investors are rational then the tangency portfolio will coincide with the market value-weighted portfolio. The arguments opposing denoting the market value-weighted portfolio as the tangency portfolio have mostly been formed as arguments denouncing the rationality of investors, e.g. highlighting different desired utilities for each investor, see e.g. [6] or [7]. However, note that the argument for equality between the tangency portfolio and the market value-weighted portfolio is also underpinned by the rather unrealistic feature that all investors share the same underlying return model. Given that this assumption is relaxed, in order to move toward a realistic setting, the market value-weighted portfolio is only a weighted average of tangency portfolios for each of the investor’s return models.

Further on, William Sharpe, in [3], formalized the capital asset pricing model (CAPM). Here, Sharpe, by utilizing the (unknown) tangency portfolio, established a relationship between the expected return for any individual constituent in the portfolio and the expected return of the tangency portfolio. In this very influential paper, Sharpe also added that *if* the market value-weighted portfolio was the tangency portfolio, then this sensitivity to the market value-weighted portfolio (where the sensitivity is estimated as the ratio of covariance between the returns of the constituent and the contemporaneous returns of the market value-weighted portfolio divided by the variance of the returns of the market value-weighted portfolio) would constitute a model to compute the expected return for each individual security. Note however, that there is an “if” in the statement and in theory, as we will display, the tangency portfolio and the market value-weighted portfolio need not be even approximately close. Later, the ratio of expected excess return above the risk-free rate, divided by the volatility of the returns of the portfolio was named the Sharpe ratio.

Today the research field of portfolio construction is extensive, spanning areas such as optimization, probability theory, statistics, and finance. Much attention is focused on factor investing and the long only version of that, *smart beta* investing see e.g. [8] or [9]. Here, the search for, and analysis of, the tangency portfolio is central. See e.g. [10, 11] for an active search for the tangency portfolio, and [12–14] or [15] for estimation and analysis of statistical properties of the tangency portfolio in both low and high dimensional settings.

In this paper, we focus on computing the tangency portfolio weights given specific underlying return models. For each setting analyzed, we find the explicit closed-form expression for the tangency portfolio weights and highlight examples where the portfolios coincide with existing smart beta products. For the most naive settings, the tangency portfolio weights are well known in the research literature. However, due to the complex optimization function and high-dimensionality of the optimization problem, with potentially thousands of constituents, explicit solutions for more general return models have not previously been formulated. The novelty of this paper is that we provide the explicit tangency portfolio weights for the return model which features a compound symmetric correlation matrix and for the return model given by the CAPM. In addition, in the special case where the return model features a compound symmetric correlation matrix and the security-specific risk-adjusted expected returns are all equal, we show that the tangency portfolio weights are proportional to the inverse of the volatilities of the constituents. For the CAPM case, we raise model consistency doubts in terms of parameter estimation. The industry standard mode of estimating the CAPM sensitivity parameters is to utilize the market value-weighted portfolio as the tangency portfolio. However, we show that the deducted explicit tangency portfolio weights under the CAPM may deviate substantially from the market value-weighted portfolio.

In the next section, “The efficient frontier and the tangency portfolio weights”, we formalize the terminology and define the tangency portfolio, as well as compute the portfolio weights for general parameter values for expected returns, risk-free rate, and covariance matrices of the returns. Followed by the section “Explicit tangency portfolio weights given underlying return models”, where we explicitly compute closed-form solutions to the optimization problem under each model. Then in section “Case study: Concentration of tangency portfolio weights”, we conduct a case study and analyze the different tangency portfolios in terms of concentration. Lastly, in section “Conclusion”, we summarize the results and discuss key findings.

## The efficient frontier and the tangency portfolio weights

First, we set up the notation and formalize the metric to be derived: the tangency portfolio weights. Furthermore, with notation in place, we compute a closed-form expression for the tangency portfolio for a general parameter setup of expected returns, risk-free rate, and covariance matrix.

There are n∈N securities in the set of investable securities and the price process for the securities is denoted by $\left\{P\left(t\right)\in R^{n}:t>0\right\}$. Future prices are unknown and the returns, $R=\left(R_{i}\right)\in R^{n}$, over some arbitrary length of Δ*t* of a future time period,

$$R_{i}=\frac{P_{i}\left(t+\Delta t\right)-P_{i}\left(t\right)}{P_{i}\left(t\right)}\;\forall i=1\ldots n$$

is a stochastic vector at each *t*. Here, we will only consider the first two moments of the returns where the expected return is given by $\mu \left(t\right)\in R^{n}$ and the covariance matrix denoted by $\Sigma \left(t\right)\in R^{n\times n}$ such that Σ_*ij*_(*t*) = Cov(*R*_*i*_, *R*_*j*_). For all *i* = 1…*n* we have that Σ_*ii*_(*t*) > 0. For brevity, *t* is dropped when it does not add any value and when appropriate, we separate the vector of volatilities, $\sigma =\left(\sigma_{i}\right)\in R^{n}$ defined by $\sigma_{i}=\sqrt{{\left(\Sigma \right)}_{ii}}$, and the correlation matrix, $C\in R^{n\times n}$, such that

$$\begin{array}{c} \Sigma =diag(\sigma )C\;diag(\sigma ) \end{array}$$ (1)

where *diag*(*σ*) denotes the volatility matrix, a diagonal matrix with vector *σ* on the diagonal. Furthermore, we assign a boldface digit to denote a vector of that number of suitable size, e.g. **1** denotes a vector ones and **0** denotes a vector zeros. Lastly, we will repeatedly utilize the proportional sign (∝) which here is defined as a relation between two vectors $w_{1},w_{2}\in R^{n}$; where it holds that *w*_1_ ∝ *w*_2_ if there exist a positive κ∈R such that *w*_1_ = *κw*_2_.

Note that all securities feature positive volatility, *σ* > **0**. Hence, they are all “risky” in some general sense since the price of the securities at the end of the period is not determined at the start of the period. In addition, throughout, we will assume that Σ is positive definite and invertible; i.e. its rows are linearly independent and therefore, the return from one security can not be exactly replicated by the return of a linear combination of other constituents.

Investors allocate their capital between the risky assets in a portfolio.

**Definition 0.1** (Portfolio). A portfolio is defined by a vector of weights, $w\left(t\right)\in R^{n}$ such that the weights sum to one and where *w*_*i*_ denotes the proportion of capital invested in security *i*. Securities in a portfolio are denoted constituents.

By the securities’ expected returns and covariances, the corresponding expected return and standard deviation of the portfolio returns are functions of the weight vector and return model parameters. Given a portfolio defined by the weights $w\in R^{n}$, the expected portfolio return is given by *μ*_*p*_(*w*) = *w*^*T*^*μ* and the standard deviation of the portfolio returns is given by $\sigma_{p}\left(w\right)=\sqrt{w^{T}\Sigma w}$.

Now, we are ready to define the efficient frontier, where in particular we have that, as in Markowitz’s original work, only non-negative weights for the constituents are considered.

**Definition 0.2** (Efficient Frontier). Markowitz’s efficient frontier is defined by the trajectory of $\left(\sigma ,\mu_{p}\left(w_{\sigma }^{*}\right)\right)$ where weights $w_{\sigma }^{*}$ are given by

$$\begin{array}{c} \begin{array}{cc} w_{\sigma }^{*}\in \underset{w\in R^{n}}{\text{argmax}}\; & w^{T}\mu \\ \text{subject}\;\text{to}:\; & w^{T}1=1\\ & w\geq 0\\ & w^{T}\Sigma w\leq \sigma^{2} \end{array} \end{array}$$ (2)

for some variable *σ* > 0 ranging over some interval.

The efficient frontier is defined by the pairs of maximal expected portfolio return for each level of volatility of the portfolio return. It is possible to state the efficient frontier by alternative optimization procedures. It can be stated either by the minimum-volatility problem, minimizing the volatility under the constraint that the expected portfolio return is at or exceeds some level, or by the mean-variance problem, maximizing the expected portfolio return, minus the variance scaled by a risk-aversion parameter. For each level of *σ* in Eq (2) there is a corresponding level of expected return in the minimum volatility-problem and a corresponding risk-aversion for the mean-variance problem such that the problems feature the same solution, see e.g. [16, 17]. Hence, a portfolio on the efficient frontier may be viewed as the portfolio that maximizes the expected return for the specific level of volatility, or equivalently as the portfolio that minimizes the volatility given at least the specific level of expected return, or again, equivalently, as the portfolio that maximizes the weighted sum of excepted return, minus scaled variance for some specific scaling parameter.

In Markowitz’s original paper, the efficient frontier was graphically represented with expected returns on the x-axis and standard deviation of the returns on the y-axis but subsequently the finance industry standard has adopted the geometrical interpretation with risk on the x-axis and expected return on the y-axis. Therefore, following industry standards, we will use this representation which has no impact on the results.

Here we consider the efficient frontier constructed with a non-negativity constraint for the weights as in complete agreement with the definition in Markowitz’s paper. Many, but not all, participants within the financial industry feature such contractual limit on the weights. However, today with the manageability of short-selling, the corresponding optimization problem without the non-negativity constraint is also of interest. It is strait-forward to show that the efficient frontier associated with the optimization problem where short selling is allowed lies at or above the efficient frontier of the original problem. But note that the additional expected return, achieved by combining the negative weights with more positive weights, features at least two caveats. First, given a portfolio features negative weights, then the downside return is theoretically unbounded and secondly, short-selling and managing such positions are associated with higher implementation costs.

In addition to the risky constituents, Tobin added a risk-free constituent to the set of investable securities, see [2]. In contrast to the risky constituents, the risk-free security features a deterministic return $r_{f}\in R$. Throughout this paper, we assume that at least one of the risky constituents features an expected return greater than *r*_*f*_. If this would not be the case, then the portfolio fully invested in the risk-free security would feature higher expected return and lower risk, since it is risk-free, than any other portfolio and the concept of the efficient frontier and the tangency portfolio rendered immaterial.

Now we are ready for the definition of the tangency portfolio weights.

**Definition 0.3** (Tangency Portfolio). The tangency portfolio is the fully-invested portfolio, with non-negative weights, among the risky constituents, that maximizes the Sharpe-ratio

$$\begin{array}{c} \begin{array}{cc} w_{tp}\in \underset{w\in R^{n}}{\text{argmax}}\; & \frac{w^{T}\mu -r_{f}}{\sqrt{w^{T}\Sigma w}}.\\ \text{subject}\;\text{to}:\; & w^{T}1=1\\ & w\geq 0 \end{array} \end{array}$$

We will also make use of the *relaxed* tangency portfolio, $w_{rtp}\in R^{n}$, a vector that sums to one, that maximizes the Sharpe ratio but not necessarily abides by the non-negativity constraint. When further distinction is needed between the two, we denote the tangency portfolio by the *proper* tangency portfolio.

**Fig 1:**
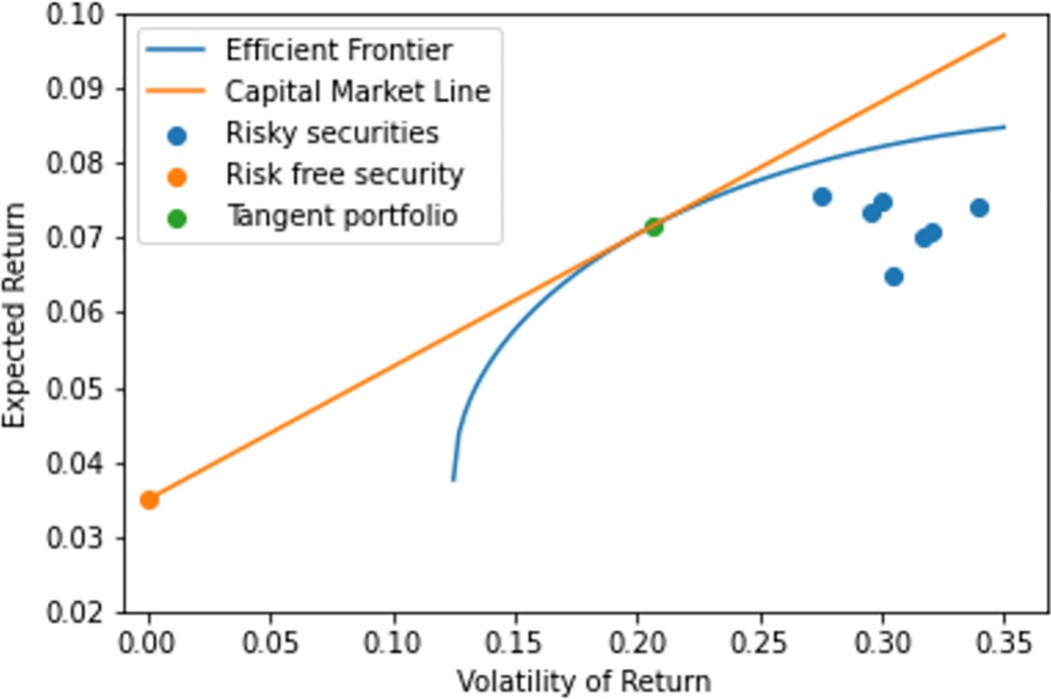
Schematic illustration of risky constituents in (*σ*, *μ*)–geometry together with the efficient frontier, the risk-free constituent, the tangency portfolio and the capital market line.

Given a risk-free security is added to the set of investable securities, then the set of portfolios featuring non-negative weights for the risky constituents and maximal Sharpe ratio is given by a continuum of portfolios such that

$$\begin{array}{c} w_{i}^{*}=\{\begin{array}{cc} 1-\xi & \;\text{if}\;i=\text{risk-free}\;\text{security}\\ \xi \;w_{tp_{i}} & \;\text{else} \end{array} \end{array}$$

for any ξ∈R such that *ξ* ≥ 0 and where *w*_*tp*_ is the tangency portfolio, as given in Definition 0.3, see e.g. [3] or [4]. Therefore, in terms of relative weights between risky constituents, i.e., all constituents except the risk-free security, the portfolios of maximal Sharpe-ratio are independent of the level of risk that is desired, and the relative weights among the risky constituents are identical to the corresponding feature of the tangency portfolio weights. The overall level of volatility that is desired for the portfolio, sets the proportion of capital invested in the risk-free constituent, but for the funds that go into risky securities, the proportions are set by the tangency portfolio weights for any rational investor. Hence, any portfolio that maximizes the Sharpe ratio for which the risk-free constituent is within the set of investable securities, then the bivariate vector of volatility and expected return of the portfolio lies on the straight-line trajectory that passes through the points (0, *r*_*f*_) and $\left(\sqrt{w_{tp}^{T}\Sigma w_{tp}},w_{tp}^{T}\mu \right)$, see Fig 1 for a schematic illustration. This line is called the Capital Market Line (CAL). Furthermore, it is from this graphical representation the naming stems. The CAL is the tangent to the efficient frontier at the point of the tangency portfolio.

Note that, in order for a portfolio to replicate the risk-return profile according to the CAL with a volatility greater than which is replicated by the tangency portfolio, the investor needs to lend capital to the risk-free return.

Note the significant implications of the feature that any point on the trajectory of maximal Sharpe ratio is attainable by a portfolio constructed of only one part the risk-free constituent and the rest allocated according the tangency portfolio weights. For example, it implies that all rational investors, independent of the desired level of risk, invest their capital proportionally equal among the risky constituents (if they share the same return model). Given an investor is risk-averse, but rational, then a large proportion of the investor’s funds go into the risk-free security but the rest is allocated proportionally to the tangency portfolio weights. Correspondingly, given that an investor is risk-seeking, but rational, then a large proportion of the investor’s funds go into the risky securities, maybe even lending to the risk-free return in order to leverage the portfolio, with proportions that are aligned with the tangency portfolio weights.

However, even though all investors allocate accordingly, their interpretation of the tangency portfolio might differ due to different underlying return models, but since the tangency portfolio is omnipresent, at least for rational investors, we will explicitly derive it given a spectra of return models. Before we proceed with that endeavour, we compute the tangency portfolio weights under a general parameter setup, i.e. for a general vector of expected returns and covariance matrix of the returns.

Given the return model, here cognizance of the parameters *μ*, *r*_*f*_ and Σ, satisfy a specific condition, then there is a closed-form expression for the relaxed tangency portfolio i.e. the fully invested portfolio, consisting only of risky assets, that maximizes the Sharpe ratio.

**Theorem 0.1** (Explicit relaxed Tangency Portfolio). *Given that the expected return μ, risk-free return r_f_, and covariance matrix* Σ *satisfies the constraint* **1**^*T*^Σ^−1^(*μ* − *r*_*f*_**1**) > 0, *then the relaxed tangency portfolio weights are given by*

$$\begin{array}{c} w_{rtp}=\frac{\Sigma^{-1}\left(\mu -r_{f}1\right)}{1^{T}\Sigma^{-1}\left(\mu -r_{f}1\right)}. \end{array}$$ (3)

See appendix for proof of Theorem 0.1.

In addition, if the relaxed tangency portfolio features only non-negative weights then it also constitutes the proper tangency portfolio.

**Lemma 0.2** (Equality of the relaxed- and the proper Tangency Portfolio). *Given that w*_*rtp*_ ≥ **0**, *i.e. all elements of the relaxed tangency portfolio are non-negative, then the proper tangency portfolio weights are identical with the relaxed tangency portfolio weights*,

$$w_{tp}=w_{rtp}.$$

See appendix for proof of Lemma 0.2.

Conversely, if for a parameter setup of *μ*, *r*_*f*_ and Σ there exists an *i* such that (Σ^−1^(*μ* − *r*_*f*_**1**))_*i*_ < 0 then then there is discrepancy between the relaxed- and the proper tangency portfolio. However, conditional that the set of constituents which feature positive weight in the proper tangency portfolio is known, denoted by $TP=\{i:w_{tp_{i}}>0\}$, then we have that the proper tangency portfolio is given by

$$w_{tp_{i}}\propto \{\begin{array}{cc} {\left(\Sigma_{TP}^{-1}\left(\mu_{TP}-r_{f}1\right)\right)}_{i} & \text{if}\;i\in TP\\ 0 & \text{else}. \end{array}$$

In order to derive closed-form expressions of the tangency portfolio weights, we will also make use of some general matrix algebra. First, since the covariance matrix is representable by the matrix product of the volatility matrix times the correlation matrix times the volatility matrix again, see Eq (1), we also have that

$$\begin{array}{c} \Sigma^{-1}=(diag(\frac{1}{\sigma }))C^{-1}(diag(\frac{1}{\sigma })). \end{array}$$ (4)

Secondly, we will repeatedly use an application of the Sherman-Morrison matrix inversion formula, see [18] or any matrix algebra reference work. The formula states that the inverse of the sum of an invertible matrix, $A\in R^{n\times n}$, and an outer product, *u v*^*T*^, where $u,v\in R^{n}$ is given by

$$\begin{array}{c} {\left(A+uv^{T}\right)}^{-1}=A^{-1}-\frac{A^{-1}uv^{T}A^{-1}}{1+v^{T}A^{-1}u}. \end{array}$$ (5)

Now we will leave the implicit framework and explicitly compute the tangency portfolio weights using specifications of the expected returns and the covariance matrix.

## Explicit tangency portfolio weights given underlying return models

Here, we will compute the closed-form expression of the tangency portfolio weights given different underlying return models, i.e. specific models for the expected return vector $\mu \in R^{n}$ and covariance matrix $\Sigma \in R^{n\times n}$.

We will see that the tangency portfolio weights may take a wide variety of shapes, spanning from equal-weighted to relatively concentrated portfolios depending on which return model the investor adheres to. In order to build the intuitive quality of the framework, we start with two naive return models: the agnostic return model and the return model with equal expected returns, for which the associated tangency portfolio weights are well-known to coincide with existing smart beta products: the 1/*n*–portfolio, respectively the minimum volatility portfolio. After that is established, we analyze more realistic return models: the return model with compound symmetric correlation matrix and the CAPM. Furthermore, we show that using the first of these models, given specific parameter limitations, then the tangency portfolio coincides with the smart beta product that features weights inversely proportional to the securities’ volatilities.

### Agnostic return model

Given that the investor features a return model that does not distinguish between any of the securities in regard to expected return and covariance structure, only acknowledging the riskiness of the investments and that the expected security returns are higher than the risk-free rate. Then for some parameters $\mu_{0},\sigma_{0},\rho \in R$ such that *μ*_0_ > *r*_*f*_ and *σ*_0_ > 0 we have that the expected return vector is given by *μ* = *μ*_0_**1** and that the covariance structure is defined by

$$\Sigma_{ij}=Cov\left(R_{i},R_{j}\right)=\sigma_{0}^{2}\{\begin{array}{cc} 1 & \text{if}\;i=j\\ \rho & \text{else} \end{array}.$$

The shape of the covariance matrix is denoted compound symmetric.

**Definition 0.4** (Compound symmetry). A square matrix, $M\in R^{n\times n}$, is denoted to feature the property of compound symmetry if the diagonal elements are all equal and all off-diagonal elements are identical

$$\begin{array}{c} M=\{\begin{array}{cc} M_{ii}=M_{jj} & \forall i,j\\ M_{ij}=M_{kl} & \forall i\neq j\;\text{and}\;\forall k\neq l \end{array}. \end{array}$$

By an application of Eq (1), we also have that the corresponding correlation matrix is compound symmetric since all volatilities are equal. Furthermore, by an application of the Sherman-Morrisson matrix inversion formula, Eq (5) with *A* = *I*(1 − *ρ*) and *u* = *ρ* **1** and *v* = **1** we have that *C* = *A* + *uv*^*T*^ and the inverse of *C* is given by

$$\begin{array}{c} {\left(C^{-1}\right)}_{ij}=\frac{1+(n-2)\rho }{\left(1-\rho \right)(1+(n-1)\rho )}\{\begin{array}{cc} 1 & \text{for}\;i=j\\ -\frac{\rho }{1+(n-2)\rho } & \text{else}\; \end{array} \end{array}$$ (6)

which also is a compound symmetric matrix. Hence, by an application of Eq (4), where *σ* ∝ **1**, also the inverse of the covariance matrix is compound symmetric. Further, by elementary linear algebra we have that the product of a compound symmetric matrix times a vector of ones is a vector of equal-sized elements. In order to see that the sign of each element is positive, we utilize that Σ (and then *C*) is positive definite. By the premise that *C* is positive definite we have that

$$0<w^{T}Cw=w^{T}(\left(1-\rho \right)w+\rho 11^{T}w)=\left(1-\rho \right)w^{T}w+\rho {\left(w^{T}1\right)}^{2},$$

for any *w* ≠ **0**. Further, by choosing $w_{i}=1/\sqrt{n}$ we have that 0 < (1 − *ρ*) + *ρn* = 1 + (*n* − 1)*ρ* which implies that

$${\left(C^{-1}1\right)}_{i}=(\frac{1+(n-2)\rho }{\left(1-\rho )(1+(n-1)\rho \right)})(1-\frac{(n-1)\rho }{1+(n-2)\rho })=\frac{1}{1+(n-1)\rho }>0$$

and then ${\left(\Sigma^{-1}1\right)}_{i}=1/\sigma_{0}^{2}{\left(C^{-1}1\right)}_{i}>0$ since *σ*_0_ > 0. Therefore, all elements of Σ^−1^(*μ* − *r*_*f*_**1**) are positive and thus **1**^*T*^Σ^−1^(*μ* − *r*_*f*_**1**) > 0. Hence, by Theorem 0.1 we have that the relaxed tangency portfolio for the agnostic return model is given by

$$w_{rtp}\propto \Sigma^{-1}1\left(\mu_{0}-r_{f}\right)\propto 1.$$

Since all elements of *w*_*rtp*_ are non-negative then by Lemma 0.2, the relaxed tangency portfolio is equal to the proper tangency portfolio, *w*_*tp*_ = *w*_*rtp*_, and the tangency portfolio weights under the agnostic return model are given by the equal-weight portfolio also known as the 1/*n*-portfolio.

### Return model with equal expected returns

Given the investor applies a return model that does not distinguish between any of the securities in regard to expected return, then by the definition of the tangency portfolio we have that

$$\begin{array}{c} \begin{array}{cc} w^{*}\in \underset{w\in R^{n}}{\text{argmax}}\; & \frac{w^{T}\mu -r_{f}}{\sqrt{w^{T}\Sigma w}}\\ \text{subject}\;\text{to}:\; & w^{T}1=1\\ & w\geq 0 \end{array}\Leftrightarrow \begin{array}{cc} w^{*}\in \underset{w\in R^{n}}{\text{argmin}}\; & w^{T}\Sigma w\\ \text{subject}\;\text{to}:\; & w^{T}1=1\\ & w\geq 0 \end{array} \end{array}$$

since *μ* ∝ **1** and we have that the numerator (*w*^*T*^*μ* − *r*_*f*_) is equal to a fixed positive number, independent of the weight vector due the sum-to-one constraint. Hence the Sharpe-ratio is maximized if and only if the variance is minimized and the tangency portfolio coincides with the minimum volatility portfolio.

In addition, since the inverse of a positive definite matrix is also positive definite, thus **1**^*T*^Σ^−1^**1** > 0 and by Theorem 0.1 we have that the relaxed tangency portfolio weights are given by

$$w_{rtp}\propto \Sigma^{-1}1.$$

Furthermore, if *w*_*rtp*_ ≥ **0**, i.e. all elements of the relaxed tangency portfolio are non-negative, then by Lemma 0.2, we have that the relaxed tangency portfolio is equal to the proper tangency portfolio.

Note however that in high dimensional settings with a large number of constituents and more complex covariance matrices, e.g. covariance matrices that feature none zero off-diagonal elements, then some of the elements of Σ^−1^**1** may be negative. For such parameter setups, the relaxed tangency portfolio and the proper tangency portfolio differ and Theorem 0.1 does not provide a closed form solution to the proper tangency portfolio weights.

Next we will analyze return models with specific covariance matrices.

### Return model with compound symmetric correlation matrix

Given that the investor adopts a return model that features identical pairwise correlations for the risky securities, then the correlation matrix is compound symmetric. By an application of combining Eq (4) with Theorem 0.1, assuming that **1**^*T*^Σ^−1^(*μ* − *r*_*f*_**1**) > 0, we have that the relaxed tangency portfolio is given by

$$\begin{array}{c} w_{rtp}\propto (diag(\frac{1}{\sigma }))C^{-1}(diag(\frac{1}{\sigma }))\left(\mu -r_{f}1\right). \end{array}$$ (7)

Further, we note that *C*^−1^ is a compound symmetric matrix proportional to a matrix with ones on the diagonal and $\delta =-\frac{\rho }{1+(n-2)\rho }\in R$ at all non-diagonal elements, see Eq (6). In order to compute the weights, we start from the end of the expression (7) and note that

$$diag\left(\frac{1}{\sigma }\right)\left(\mu -r_{f}1\right)=\left(\frac{\mu -r_{f}1}{\sigma }\right)\in R^{n},$$

where the division operation is defined element-wise. By multiplying *C*^−1^ from the left, then for the first element of $C^{-1}\left(\frac{\mu -r_{f}1}{\sigma }\right)$ we have that

$$\begin{array}{c} (C^{-1}\left(\frac{\mu -r_{f}1}{\sigma }\right){)}_{1}=\frac{1+(n-2)\rho }{\left(1-\rho \right)(1+(n-1)\rho )}(\frac{\mu_{1}-r_{f}}{\sigma_{1}}+\delta \sum_{i=2}^{n}\frac{\mu_{i}-r_{f}}{\sigma_{i}}). \end{array}$$

By further exploring the second term on the right hand side, we have that

$$\begin{array}{ccc} \frac{\mu_{1}-r_{f}}{\sigma_{1}}+\delta \sum_{i=2}^{n}\frac{\mu_{i}-r_{f}}{\sigma_{i}} & = & \left(1-\delta \right)\frac{\mu_{1}-r_{f}}{\sigma_{1}}+\delta n\bar{{\left\{\frac{\mu_{i}-r_{f}}{\sigma_{i}}\right\}}_{j=1}^{n}}\\ & = & (\frac{1+(n-1)\rho }{1+(n-2)\rho })\frac{\mu_{1}-r_{f}}{\sigma_{1}}-\frac{\rho }{1+(n-2)\rho }n\bar{{\left\{\frac{\mu_{i}-r_{f}}{\sigma_{i}}\right\}}_{j=1}^{n}}, \end{array}$$ (8)

where at the first equal sign, we added and subtracted the entity $\delta \frac{\mu_{1}-r_{f}}{\sigma_{1}}$ as well as utilized the notation $\bar{x}$ to denote the mean of vector *x*. At the second equal sign, we inserted the definition of *δ*. Then by factoring out the common term of the two addends, $\frac{1}{1+(n-2)\rho }$, and rearranging, we have that

$$\begin{array}{ccc} (C^{-1}\left(\frac{\mu -r_{f}1}{\sigma }\right){)}_{1} & = & \frac{1}{\left(1-\rho \right)(1+(n-1)\rho )}(\left(1-\rho \right)\frac{\mu_{1}-r_{f}}{\sigma_{1}}+n\rho \left(\frac{\mu_{1}-r_{f}}{\sigma_{1}}-\bar{{\left\{\frac{\mu_{i}-r_{f}}{\sigma_{i}}\right\}}_{j=1}^{n}}\right))\\ & = & \frac{1}{\left(1+(n-1)\rho \right)}(\frac{\mu_{1}-r_{f}}{\sigma_{1}}+\frac{n\rho }{1-\rho }\left(\frac{\mu_{1}-r_{f}}{\sigma_{1}}-\bar{{\left\{\frac{\mu_{i}-r_{f}}{\sigma_{i}}\right\}}_{j=1}^{n}}\right)), \end{array}$$

where at the second equal sign, we factored in the term $\frac{1}{1-\rho }$ to the two addends. All subsequent elements of $C^{-1}\left(\frac{\mu -r_{f}1}{\sigma }\right)$ may be computed analogously. Further, by multiplying by $diag(\frac{1}{\sigma })$ from the left, we have that by Theorem 0.1, the relaxed tangency portfolio weights are given by

$$\begin{array}{c} w_{rtp}\propto \frac{1}{\sigma }.(\frac{\mu -r_{f}1}{\sigma }+\frac{n\rho }{1-\rho }\left(\frac{\mu -r_{f}1}{\sigma }-\bar{{\left\{\frac{\mu_{j}-r_{f}}{\sigma_{j}}\right\}}_{j=1}^{n}}1\right)), \end{array}$$ (9)

where the dot (.) denotes element-wise multiplication and if the theorem premise inequality

$${\left(\frac{1}{\sigma }\right)}^{T}(\frac{\mu -r_{f}1}{\sigma }+\frac{n\rho }{1-\rho }\left(\frac{\mu -r_{f}1}{\sigma }-\bar{{\left\{\frac{\mu_{j}-r_{f}}{\sigma_{j}}\right\}}_{j=1}^{n}}1\right))>0$$

is attained.

In addition, since *σ* > **0**, if we have that

$$\frac{\mu_{i}-r_{f}}{\sigma_{i}}+\frac{n\rho }{1-\rho }\left(\frac{\mu_{i}-r_{f}}{\sigma_{i}}-\bar{{\left\{\frac{\mu_{j}-r_{f}}{\sigma_{j}}\right\}}_{j=1}^{n}}\right)\geq 0$$

for all *i* then by Lemma 0.2, Eq (9) also defines the proper tangency portfolio.

In order to build intuition of the implications of Eq (9), we here will highlight a couple of examples. First, by restricting the return model parameters, making the model identical to the agnostic return model by fixing *μ* ∝ **1** and *σ* ∝ **1**, then we have that Eq (9) replicates the result that the relaxed tangency portfolio, under this return model, is equal to the equal-weighted portfolio.

Returning to the general case: if, for each security, we denote the ratio of expected return minus the risk-free return, both divided by the volatility, for the security-specific risk-adjusted expected return, then we have that the weight of each constituent is inversely proportional to the volatility times the sum of the risk-adjusted expected return and scaled deviation of risk-adjusted expected return from the mean of the same vector, where the scaling is proportional to the breadth of the investable universe.

For the case where *n* is large, then the relaxed tangency portfolio may exhibit extreme proportional differences in weights between constituents even where there are only small deviances in expected return and/or volatilities. In order to illustrate that property we design Example 0.1.

**Example 0.1**. For the compound symmetric covariance matrix with *n* = 1508 securities (the number of constituents in MSCI World as of 31jan2023), with *ρ* = 0.25 and volatilities *σ*_*i*_ = 0.3 for all constituents and *μ*_*i*_ = *r*_*f*_ + 0.05 for all constituents except the three last which feature *μ*_*i*_ = *r*_*f*_ + 0.07 then we have that *w*_*rtp*_ ≥ **0** where *w*_*rtp*_ is given by Eq (9), i.e. all elements of the relaxed tangency portfolio are non-negative. Hence, by Lemma 0.2, the relaxed tangency portfolio coincides with the proper tangency portfolio, *w*_*tp*_ = *w*_*rtp*_. Furthermore, we have that $w_{tp_{n}}/w_{tp_{1}}>500$ i.e. the last three constituents holds each more than 500 times more weight than any other individual constituent, and the tangency portfolio allocates more than 50% of the weight to the three last constituents.

By the example we have that even in the case of low complexity, with a compound symmetric correlation matrix and equal volatilities, small deviations in expected return may constitute large differences in weights of the tangency portfolio.

Another example, for the special case where the security-specific risk-adjusted expected returns are all equal, i.e. where we have that $\frac{\mu -r_{f}1}{\sigma }\propto 1$, then the second term in the Eq (9) is nullified, i.e. there are no deviations from cross-sectional mean risk-adjusted expected return. Furthermore, since the security-specific risk-adjusted expected returns are all equal, then the first term in Eq (9) is proportional to **1**. In addition, since all the volatilities are positive, then $1^{T}\frac{1}{\sigma }>0$, thus the premise of Theorem 0.1 is attained and the relaxed tangency portfolio is given by the inverse-volatility portfolio i.e. the portfolio with weights proportional to the reciprocal of the volatilities,

$$w_{rtp}\propto \frac{1}{\sigma }.$$

**Fig 2:**
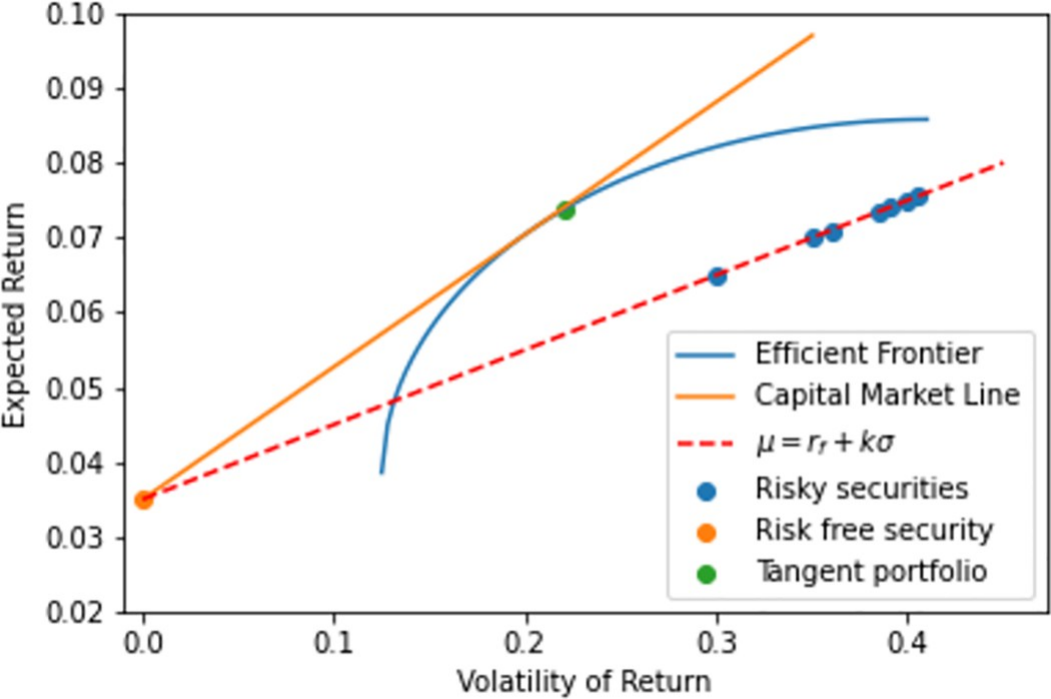
Schematic illustration of risky constituents in (*σ*, *μ*)–geometry where the correlation matrix is compound symmetric and all constituents feature equal expected risk adjusted return, *μ* = *r*_*f*_ + *kσ*. Here the tangency portfolio weights are given by the inverse-volatility portfolio.

In addition, in this special case, since all volatilities are positive, then by Lemma 0.2, the portfolio also constitutes the proper tangency portfolio. Fig 2 displays a schematic illustration where the security-specific risk-adjusted expected returns are all equal.

### Capital asset pricing model

Sharpe introduced the Capital Asset Pricing Model (CAPM) in his seminal paper [3]. Here, Sharpe ingeniously, implicitly utilizes the tangency portfolio as a reference portfolio to construct an equilibrium model for the expected return for each security. The model is defined by the sensitivities, also known as betas (*β*), which are given by the covariance between the security-specific return and the return of the reference portfolio, divided by the variance of the portfolio return. However, Sharpe does not explicitly define the weights of the tangency portfolio but notes that if the market value-weighted portfolio is the tangency portfolio, then these sensitivities can be estimated from historical return data. Later, perhaps partly due to the consequences of the separation theorem and the argumentation of market equilibrium both by Sharpe in [3] and Lintner in [5], the general approach in the industry has been to utilize the market value-weighted portfolio as the reference portfolio for the CAPM.

Here, we will reverse engineer Sharpe’s work and assume that we have estimates of the model parameters and work ourselves backward, and deduce the tangency portfolio weights given this return model.

First, we introduce the most commonly used asset pricing model in the finance industry: the CAPM. The CAPM, with the tangency portfolio as reference portfolio, states that the returns, $\left(R_{i}\right)\in R^{n}$, are governed by

$$\begin{array}{c} R_{i}=r_{f}+\beta_{i}\left(R_{tp}-r_{f}\right)+\varepsilon_{i}\;\forall i=1\ldots n, \end{array}$$ (10)

where $\beta =\left(\beta_{i}\right)\in R^{n}$ is a vector of parameters and $\varepsilon =\left(\varepsilon_{i}\right)\in R^{n}$ is a stochastic vector, independent of *R*_*tp*_, which features zero expected return for each element. The elements of *ε* are mutually independent and the variance of each element, $\text{Var}\left(\varepsilon_{i}\right)=\sigma_{I_{i}}^{2}$, is denoted by the idiosyncratic variance. Furthermore, $R_{tp}\in R$ is a stochastic variable, the contemporaneous return of the tangency portfolio, with expected value $\mu_{tp}\in R$ such that *μ*_*tp*_ > *r*_*f*_, and variance $\sigma_{tp}^{2}\in R$.

By the CAPM model, Eq (10), we have that the vector of expected excess returns, expected return minus the risk-free return, is proportional to *β*,

$$\mu -r_{f}1\propto \beta ,$$

since the market risk premium, the expected return of the tangency portfolio minus the risk-free return, is positive. The covariance matrix of the security returns is given by

$$\Sigma =\beta \beta^{T}\sigma_{tp}^{2}+diag\left(\sigma_{I}^{2}\right).$$

In order to derive the tangency portfolio weights for this return model we first note that by another application of the Sherman-Morrison matrix inversion formula, Eq (5) with $A=diag(\sigma_{I}^{2})$ and *u* = *v* = *σ*_*tp*_*β*, we have that

$$\begin{array}{c} \Sigma^{-1}=diag\left(1/\sigma_{I}^{2}\right)-\frac{\sigma_{tp}^{2}}{1+\sigma_{tp}^{2}\kappa }\left(\frac{\beta }{\sigma_{I}^{2}}\right){\left(\frac{\beta }{\sigma_{I}^{2}}\right)}^{T}, \end{array}$$ (11)

where $\kappa ={\left(\frac{\beta }{\sigma_{I}}\right)}^{T}\left(\frac{\beta }{\sigma_{I}}\right)$. Then by invoking Theorem 0.1, where for now we assume that the theorem premise is attained, combined with Eq (11), we have

$$\begin{array}{ccc} w_{rtp} & \propto & (diag\left(1/\sigma_{I}^{2}\right)-\frac{\sigma_{tp}^{2}}{1+\sigma_{tp}^{2}\kappa }(\frac{\beta }{\sigma_{I}^{2}})(\frac{\beta }{\sigma_{I}^{2}}{)}^{T})\beta \\ & = & \left(\frac{\beta }{\sigma_{I}^{2}})-\frac{\sigma_{tp}^{2}}{1+\sigma_{tp}^{2}\kappa }(\frac{\beta }{\sigma_{I}^{2}})(\frac{\beta }{\sigma_{I}}{)}^{T}(\frac{\beta }{\sigma_{I}}\right)\\ & = & (\frac{\beta }{\sigma_{I}^{2}})\frac{1}{1+\sigma_{tp}^{2}{\left(\frac{\beta }{\sigma_{I}}\right)}^{T}\left(\frac{\beta }{\sigma_{I}}\right)} \end{array}$$ (12)

where at the second equal sign the definition of *κ* is utilized.

Returning to the premise of Theorem 0.1, if $\beta^{T}\left(\frac{1}{\sigma_{I}^{2}}\right)>0$, then by Eq (12) we have that the relaxed tangency portfolio weights given the CAPM return model are given by weights proportional to beta, divided by the idiosyncratic variance,

$$\begin{array}{c} w_{rtp}\propto \beta /\sigma_{I}^{2}. \end{array}$$ (13)

In addition, if we have that *β* ≥ **0**, i.e. all betas are non-negative, then since $\sigma_{I}^{2}$ is a vector of positive elements, by Lemma 0.2, we have that also the proper tangency portfolio is given by Eq (13).

The industry standard adoption of the CAPM model is to utilize the market value-weighted portfolio as the reference portfolio in Eq (10), i.e. estimates of beta and idiosyncratic variance are computed as the sample covariance between each security return and the return of the market value-weighted portfolio and unexplained variability from that specific model. Furthermore, the logical chain of arguments in deriving the tangency portfolio weights for that return model is identical, only noting that the betas and idiosyncratic variance applied are given in reference to the market value-weighted portfolio. However, for where this approach with the market value-weighted portfolio is applied as the reference portfolio, it should be noted that since the tangency portfolio is deducable from the return model, Eq (13), there are inherent inconsistencies in the model setup if the tangency portfolio implied by the utilized return model is far from the market value-weighted portfolio.

## Case study: Concentration of tangency portfolio weights

There are many aspects of risks in terms of portfolio construction. One dimension to consider is concentration risk, i.e. the amount of weight that is distributed to a single or a small set of constituents of the portfolio.

One way of displaying the concentration of the weights is by the Lorenz curve which was originally introduced as a graphical representation of the distribution of income (or of wealth) representing the inequality of wealth distribution [19]. Here, the Lorenz curve represents the proportion of the total weight that is cumulatively aggregated by the constituents, where the constituents in the portfolio are ordered in ascending individual weight size. The two extreme cases, in terms of concentration, are the equal-weighted portfolio, that maximizes dispersion of the weight, and the portfolio consisting of investment in one single constituent. These portfolios define the upper and lower bounds of the Lorenz curve for all portfolios, where the Lorenz curve of the equal-weighted portfolio is depicted by the straight line (*x*, *y*) such that *y*(*x*) = *x*/*n* for all *x* = 1, 2, …, *n*, and the Lorenz curve of the portfolio consisting of investment in one single constituent is represented by the function that takes the value zero for *x* = 1, 2, …, *n* − 1 and one at *x* = *n*. In addition, the Gini coefficient, introduced by the Italian statistician Corrado Gini, see e.g. [20], quantifies this concentration property. The Gini coefficient is the ratio of the area that falls between the Lorenz curve of the equal-weighted portfolio and the Lorenz curve of the measured portfolio, divided by the area under the Lorenz curve of the equal-weighted portfolio. Hence, the equal-weighted portfolio features a Gini coefficient equal to zero, while the highly concentrated portfolio in the example above, features a Gini coefficient equal to one.

In order to give realistic examples of concentration measures, we compute the respective tangency portfolio weights with security-specific properties estimated from real-world data. Here, we use the securities defined in MSCI World, that, as of date 31-January-2023, consist of 1508 constituents, as the investable universe. The return data associated with the securities stems from Refinitiv Datastream.

The tangency portfolios considered here are: the equal-weight portfolio, the minimum-volatility portfolio, the compound-symmetry example portfolio (see Example 0.1), the inverse-volatility portfolio, the CAPM-tangency portfolio, and the market value-weighted portfolio.

**Fig 3:**
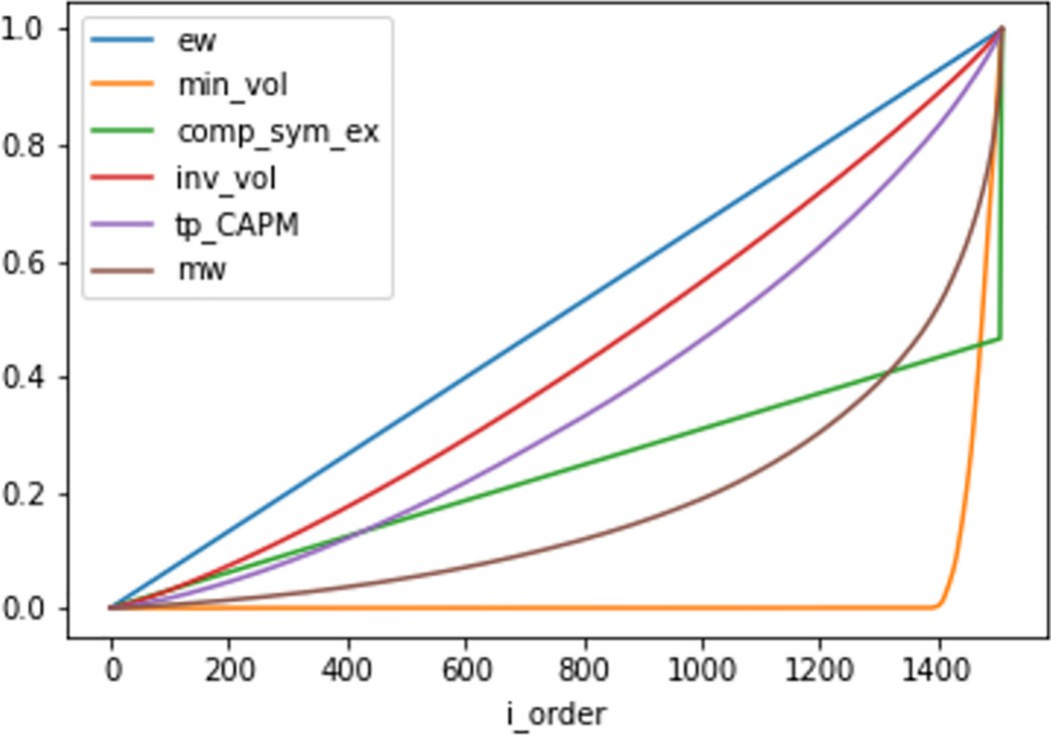
The Lorenz curves for six portfolios; the equal-weight portfolio (ew), the minimum-volatility portfolio (min_vol), the portfolio from Example 0.1 with the compound-symmetric correlation matrix (comp_sym_ex), the inverse-volatility portfolio (inv_vol), the CAPM-tangency portfolio (tp_CAPM) and the market value-weighted portfolio (mw).

The Lorenz curves of the respective tangency portfolio are depicted in Fig 3 and the associated Gini coefficients are displayed in Table 1.

**Table 1:** The Gini coefficients of weight distribution of the (tangency) portfolios considered in Fig 3.

Portfolio	Gini coefficient
equal-weighted	0
minimum-volatility	0.95
compound symmetry example	0.53
inverse-volatility	0.16
CAPM-tangency portfolio	0.29
market value-weighted	0.65

Before we examine the results, we disclose some additional model choices we applied in order to construct the minimum-volatility portfolio, the inverse-volatility portfolio, and the CAPM tangency portfolio. Note however, this a schematic illustration, hence the specific model choices are not essential for the analysis. The inverse-volatility portfolio is constructed by the volatilities estimated from one-week return periods measured using a historical window of three years. The covariance matrix of the minimum-volatility portfolio is estimated using the first fifty principal components (which capture about 70% of the total variability in the sample) supplied with idiosyncratic risk in order for the respective volatilities of the model to coincide with the empirical volatilities of each element, all based on the same length of return period and historical window as above. In addition, a lower limit of annualized volatility of 0.3 was imputed to the estimated covariance matrix and following MSCI methodology, see [21], an individual weight maximum of 1.5% was imposed in the optimization procedure. Lastly, the parameters for the CAPM-tangency portfolio were estimated with return data based on the same length of return period and historical window as above and the market value-weighted portfolio was used as the reference portfolio.

As seen in Fig 3 and Table 1, depending on which return model was utilized, we have that the concentration of the tangency portfolios may be both more or less profound compared to the market value-weighted portfolio.

The result that the Lorenz curve of the tangency portfolio extracted from the CAPM model is distant from the Lorenz curve of the market value-weighted portfolio, highlights some model estimation incoherence in the CAPM framework. The theoretical framework for CAPM implicitly uses the assumption that the market value-weighted portfolio is the tangency portfolio. Here, by adopting the industry standard techniques to estimate *β* and idiosyncratic volatility, we note that the greatest relative difference between the security-specific quotients $\beta /\sigma_{I}^{2}$ for any pair of constituents is about 20. In contrast, the ratio of maximal proportional weight difference in the market value-weighted portfolio, computed as the largest weight divided by the smallest weight, is much larger in MSCI World as of 31-Jan-2023 where that ratio is above 1400. This further illustrates that the market value-weighted portfolio is much more concentrated than the analytically-derived tangency portfolio, given the CAPM return model as verified in Fig 3.

### Deviance between the relaxed tangency portfolio and the proper tangency portfolio

For one of the return models in the example above, the return model featuring equal expected returns and with a covariance matrix estimated by the principal components, the proper tangency portfolio weights differ from the corresponding relaxed tangency portfolio weights. In regard to concentration, the portfolios differ substantially. However, for portfolios with negative weights the Gini coefficient is not an informative metric, instead we display the concentration difference of the portfolios by the number of constituents featuring a weight deviating from zero. The proper tangency portfolio i.e. the minimum-volatility portfolio, which features the non-negativity constraint, allocates weight to a small proportion of the constituents, see Fig 3. In this specific sample, the weights of the minimum-volatility portfolio are such that there are only 142 constituents (out of 1508) with a weight above 0.001*bps*. Since the optimal weights here are found by a numerical inner point optimization algorithm, we consider weights less than that threshold to be of negligible magnitude and don’t constitute a meaningful mass (and are rather a consequence of the applied levels of the stopping criteria for the numerical algorithm). In contrast, by utilizing the same covariance matrix that we used to compute the minimum-volatility portfolio, applying the same optimization procedure, only ignoring the non-negativity constraints, we find that $|w_{rtp_{i}}|>0.001bps$ for all *i*; i.e. a non-negligible portion of the capital, either positive or negative, should be invested in every one of the constituents in order to maximize the Sharpe-ratio of the portfolio if short-selling is allowed. Hence, in this example, the relaxed tangency portfolio deviates substantially from the proper tangency portfolio.

## Conclusion

Here we have computed closed-form expressions for the tangency portfolio weights given a variation of return models, see Table 2 for a summary.

**Table 2:** The summary of explicit relaxed tangency portfolios given return models.

Return model	Descriptive name of the tangency portfolio	Weight proportions for the relaxed tangency portfolio weights
Equal expected returns and Compound symmetric covariance matrix	equal-weighted, 1/*n*	**1**
Equal expected returns	minimum-volatility	Σ^−1^**1**
Compound symmetric correlation matrix + equal expected risk-adjusted excess return for all securities	inverse-volatility	$\left(1/\sigma \right).(\frac{\mu -r_{f}1}{\sigma }+\frac{n\rho }{1-\rho }\left(\frac{\mu -r_{f}1}{\sigma }-\bar{{\left\{\frac{\mu_{j}-r_{f}}{\sigma_{j}}\right\}}_{j=1}^{n}}1\right))$ 1/*σ*
CAPM	beta-over-idiosyncratic variance	$\beta /\sigma_{I}^{2}$

The dot (.) and division (/) signs denotes element-wise multiplication and division respectively.

Depending on the return model, the tangency portfolio weights take a diverse set of shapes; from very diversified to highly concentrated portfolios. We have also related the derived tangency portfolio weights to the market value-weighted portfolio, where for the tangency portfolio weights associated with the return models presented in this paper, there are considerable discrepancies in terms of concentration of the portfolios. Both examples of tangency portfolios that are less concentrated and examples of tangency portfolios that feature higher concentration have been displayed.

Lastly, investment managers market off-the-shelf, rules-based, alternatively-weighted index funds such as equal-weight, minimum-volatility and inverse-volatility indices as simple, low-cost ways to gain exposure to market risk premia. However, here we have supplied an additional *raison d’être*. These indices are all, respectively, the actual tangency portfolio weights associated with, for each set up, (possibly) reasonable return models.

## Appendix

### Proof of Theorem 0.1 (Explicit relaxed Tangency Portfolio)

*Proof*. In order to prove Theorem 0.1 we will first modify the objective function such that it becomes independent of the magnitude of the argument. Then we will remove the sum-to-one constraint, featuring an unconstrained optimization problem, and note that due to the objective function being independent of the magnitude of the argument, we will retrieve the solution to the original problem by the solution to this simpler problem.

First we note that for *w*^*T*^**1** = 1 then *w*^*T*^*μ* − *r*_*f*_ = *w*^*T*^(*μ* − *r*_*f*_**1**) and the transformed objective function of the Sharpe-ratio optimization problem,

$$\begin{array}{c} g\left(w\right)=\frac{w^{T}\left(\mu -r_{f}1\right)}{\sqrt{w^{T}\Sigma w}}, \end{array}$$ (14)

is independent of the magnitude of *w*^*T*^**1**, i.e. *g*(*w*°) = *g*(*κw*°) for any κ∈R:κ>0 and if $w^{\circ }\in R^{n}$ is a point of maximum to the function *g*, then so is *κw*° if *κ* > 0. Hence we have an unconstrained optimization problem of maximizing *g*, as defined by Eq (14), that is independent of the magnitude of the argument. Therefore, to avoid a multiplicity of solutions to the unconstrained maximization problem of objective function *g*, we simplify the optimization problem by imposing the constraint *w*^*T*^*Σw* = 1. The modified optimization problem becomes

$$\begin{array}{c} \begin{array}{cc} w^{*}\in \underset{w\in R^{n}}{\text{argmax}}\; & \frac{w^{T}\left(\mu -r_{f}1\right)}{\sqrt{w^{T}\Sigma w}}\\ \text{subject}\;\text{to}:\; & w^{T}\Sigma w=1 \end{array}\Leftrightarrow \begin{array}{cc} w^{*}\in \underset{w\in R^{n}}{\text{argmax}}\; & w^{T}\left(\mu -r_{f}1\right)\\ \text{subject}\;\text{to}:\; & w^{T}\Sigma w=1 \end{array} \end{array}$$ (15)

Furthermore, if we find *w*°, a global unique solution to problem (15), and rescale the solution by the factor *κ* = (**1**^*T*^*w*°)^−1^ where κ∈R is positive, then the rescaled vector is the relaxed tangency portfolio weights since it maximizes the Sharpe ratio and sums to one.

We relax the constraint of optimization problem (15) to feature an inequality constraint,

$$w^{T}\Sigma w\leq 1.$$

This technicality makes the feasible set convex but, as will be displayed, does not alter the optimal solution. By standard optimization techniques, we have that the Lagrangian function associated with the optimization problem is given by

$$\begin{array}{c} L\left(w,\lambda \right)=-w^{T}\left(\mu -r_{f}1\right)+\lambda \left(w^{T}\Sigma w-1\right) \end{array}$$

with the KKT conditions

$$\begin{array}{ccc} -(\mu -r_{f}1)+2\lambda \Sigma w & = & 0 \end{array}$$ (16)

$$\begin{array}{ccc} \lambda & \geq & 0\\ \lambda (w^{T}\Sigma w-1) & = & 0\\ w^{T}\Sigma w & \leq & 1 \end{array}$$ (17)

see e.g. [22].

If we assume an optimal vector *w*° satisfies (*w*°)^*T*^Σ*w*° < 1 then λ° = 0, due to Eq (17), which by Eq (16) leads to a contradiction since *μ*_*i*_ − *r*_*f*_ > 0 for at least one *i* = 1…*n*. Hence for a point $\left(w^{\circ },\lambda^{\circ }\right)\in R^{n+1}$ to uphold the KKT conditions, then λ° > 0 and by Eq (17) we have (*w*°)^*T*^Σ*w*° = 1 and thus the relaxation of the sum-to-one constraint had no impact on the solution.

Since the function to be maximized is concave and the feasible set is convex, the global maximum is realized at a KKT point, see e.g. [22]. The unique KKT point, the vector (*w*°, λ°) that solves the system of Eqs (16) and (17), is given by

$$\begin{array}{ccc} w^{\circ } & = & \frac{\Sigma^{-1}\left(\mu -r_{f}1\right)}{\sqrt{{\left(\mu -r_{f}1\right)}^{T}\Sigma^{-1}\left(\mu -r_{f}1\right)}}\\ \lambda^{\circ } & = & \frac{1}{2}\sqrt{{\left(\mu -r_{f}1\right)}^{T}\Sigma^{-1}\left(\mu -r_{f}1\right)}. \end{array}$$

Hence, *w*° is the global maximizer of optimization problem (15).

Furthermore, since **1**^T^*w*° ∝ **1**^*T*^Σ^−1^(*μ* − *r*_*f*_**1**) > 0 by an application of the theorem premise then *κ* = (**1**^*T*^*w*°)^−1^ > 0, and the relaxed tangency portfolio weights are well defined, unique and given by

$$w_{rtp}=\kappa w^{\circ }=\frac{\Sigma^{-1}\left(\mu -r_{f}1\right)}{1^{T}\Sigma^{-1}\left(\mu -r_{f}1\right)},$$

since $w_{rtp}\in R^{n}$ sum to one and maximizes the Sharpe ratio.

### Proof of Lemma 0.2 (Equality of the relaxed- and the proper Tangency Portfolio)

*Proof*. If *w*_*rtp*_ ≥ **0** then adding element-wise non-negativity restrictions to the variables of the optimization procedure does not change the solution, since all those inequality constraints are inactive. Further, such optimization procedure is identical to Definition 0.3 and there is equality between the relaxed- and the proper tangency portfolio.

## References

[1] Markowitz H. Portfolio Selection. The Journal of Finance. 1952;7(1):77–91. doi: 10.1111/j.1540-6261.1952.tb01525.x

[2] Tobin J. Liquidity preference as behavior towards risk. The review of economic studies. 1958;25(2):65–86. doi: 10.2307/2296205

[3] Sharpe WF. Capital asset prices: A theory of market equilibrium under conditions of risk. The journal of finance. 1964;19(3):425–442. doi: 10.2307/2977928

[4] Lintner J. Security prices, risk, and maximal gains from diversification. The journal of finance. 1965;20(4):587–615. doi: 10.2307/2977249

[5] Lintner J. The Valuation of Risk Assets and the Selection of Risky Investments in Stock Portfolios and Capital Budgets. The Review of Economics and Statistics. 1965;47(1):13–37. doi: 10.2307/1924119

[6] Warren GJ. Choosing and Using Utility Functions in Forming Portfolios. Financial Analysts Journal. 2019;75(3):39–69. doi: 10.1080/0015198X.2019.1618109

[7] Kahneman D, Tversky A. Prospect theory: An analysis of decision under risk. In: Handbook of the fundamentals of financial decision making: Part I. World Scientific; 2013. p. 99–127.

[8] Bessler W, Taushanov G, Wolff D. Factor investing and asset allocation strategies: a comparison of factor versus sector optimization. Journal of Asset Management. 2021;22(6):488–506. doi: 10.1057/s41260-021-00225-1

[9] Martellini L. Toward the design of better equity benchmarks: Rehabilitating the tangency portfolio from modern portfolio theory. The Journal of Portfolio Management. 2008;34(4):34–41. doi: 10.3905/jpm.2008.709978

[10] Muhinyuza S, Bodnar T, Lindholm M. A test on the location of the tangency portfolio on the set of feasible portfolios. Applied Mathematics and Computation. 2020;386:125519. doi: 10.1016/j.amc.2020.125519

[11] Feng G, Jiang L, Li J, Song Y. Deep Tangency Portfolios. SSRN. 2023; 10.2139/ssrn.3971274.

[12] Alfelt G, Mazur S. On the mean and variance of the estimated tangency portfolio weights for small samples. Modern Stochastics: Theory and Applications. 2022;9(4):453–482.

[13] Bodnar T, Mazur S, Podgórski K, Tyrcha J. Tangency portfolio weights for singular covariance matrix in small and large dimensions: Estimation and test theory. Journal of Statistical Planning and Inference. 2019;201:40–57. doi: 10.1016/j.jspi.2018.11.003

[14] Farrukh Javed SM, Ngailo E. Higher order moments of the estimated tangency portfolio weights. Journal of Applied Statistics. 2021;48(3):517–535. doi: 10.1080/02664763.2020.1736523 35706539 PMC9041990

[15] Karlsson S, Mazur S, Muhinyuza S. Statistical inference for the tangency portfolio in high dimension. Statistics. 2021;55(3):532–560. doi: 10.1080/02331888.2021.1951730

[16] Krokhmal P, Palmquist J, Uryasev S. Portfolio optimization with conditional value-at-risk objective and constraints. Journal of risk. 2002;4:43–68. doi: 10.21314/JOR.2002.057

[17] Rockafellar RT, Uryasev S. Conditional value-at-risk for general loss distributions. Journal of banking & finance. 2002;26(7):1443–1471. doi: 10.1016/S0378-4266(02)00271-6

[18] Sherman J, Morrison WJ. Adjustment of an inverse matrix corresponding to a change in one element of a given matrix. The Annals of Mathematical Statistics. 1950;21(1):124–127. doi: 10.1214/aoms/1177729893

[19] Lorenz MO. Methods of measuring the concentration of wealth. Publications of the American statistical association. 1905;9(70):209–219. doi: 10.1080/15225437.1905.10503443

[20] Ceriani L, Verme P. The origins of the Gini index: extracts from Variabilità e Mutabilità (1912) by Corrado Gini. The Journal of Economic Inequality. 2012;10:421–443. doi: 10.1007/s10888-011-9188-x

[21] MSCI. MSCI minimum volatility indexes methodology. MSCI; 2017. Available from: https://www.msci.com/eqb/methodology/meth_docs/MSCI_Minimum_Volatility_Methodology_Sep2017.pdf.

[22] Andréasson N, Evgrafov A, Patriksson M. An Introduction to Continuous Optimization. Professional Publishing Svc.; 2005. Available from: https://books.google.se/books?id=dxdCHAAACAAJ.

